# Incidence of Bone Metastases and Skeletal-Related Events in Patients With *EGFR*-Mutated NSCLC Treated With Osimertinib

**DOI:** 10.1016/j.jtocrr.2023.100513

**Published:** 2023-04-03

**Authors:** Anita J.W. M. Brouns, Ard van Veelen, G. D. Marijn Veerman, Christi Steendam, Safiye Dursun, Cor van der Leest, Sander Croes, Anne-Marie C. Dingemans, Lizza E.L. Hendriks

**Affiliations:** a Department of Respiratory Medicine, Zuyderland, The Netherlands; b Department of Respiratory Medicine, Maastricht University Medical Center+, Maastricht, The Netherlands; c GROW—School for Oncology and Reproduction, Universiteitssingel 40, Maastricht, The Netherlands; d Department of Clinical Pharmacy and Toxicology, Maastricht University Medical Center+, Maastricht, The Netherlands; e CARIM School for Cardiovascular Disease, Maastricht University Medical Center+, Maastricht, The Netherlands; f Department of Respiratory Medicine, Erasmus MC Cancer Institute, University Medical Center Rotterdam, Rotterdam, The Netherlands; g Department of Medical Oncology, Erasmus MC Cancer Institute, University Medical Center Rotterdam, Rotterdam, The Netherlands; h Department of Respiratory Medicine, Amphia Hospital Breda, Breda, The Netherlands

**Keywords:** Bone metastases related outcomes, Tyrosine kinase inhibitor, Lung adenocarcinoma, Bone targeted agents, Solid tumors

## Abstract

**Introduction:**

Bone metastases are frequent in patients with *EGFR*-mutated (*EGFR+*) NSCLC. Skeletal-related events (SREs) are common in these patients; however, no data on SRE in osimertinib-treated patients are reported. We investigated the development of bone metastases and SREs in patients with *EGFR+* NSCLC treated with osimertinib.

**Methods:**

This is a retrospective multicenter cohort study that included patients with metastatic *EGFR+* NSCLC who were treated with osimertinib between February 2016 and September 2021. Demographics, bone metastases-related outcomes, SREs, treatment efficacy, and overall survival (OS) were collected.

**Results:**

In total, 250 patients treated with osimertinib (43% first line) were included. Of the patients, 51% had bone metastases at initiation of osimertinib. Furthermore, 16% of the patients with bone metastases used bone-targeted agents. Median follow-up from initiation of osimertinib was 23.4 months (95% confidence interval [CI]: 19.9–26.9 mo). During osimertinib treatment, 10% developed new bone metastases or bone progression. Of the patients with bone metastases, 39% had more than or equal to one SREs: 28% developed first SRE before osimertinib treatment, 1% after, and 11% during. Median OS post-bone metastasis was 30.8 months (95% CI: 21.9–39.7). Median OS after first SRE was 31.1 months (95% CI: 15.8–46.5).

**Conclusions:**

Bone metastases and SREs are frequent before and during treatment with osimertinib in *EGFR+* NSCLC. Because of these findings and the long OS post-bone metastases, we advocate prescription of bone-targeted agents in these patients and recommend adding bone-specific end points in clinical trials.

## Introduction

Bone metastases occur in 30% to 60% of patients with advanced NSCLC.^1^^,^^2^ Patients with bone metastases are at risk for skeletal-related events (SREs), with subsequently a possible negative impact on quality of life (QoL) and overall survival (OS).^3^, ^4^, ^5^ The term SRE is a composite end point consisting of pathologic fracture, spinal cord compression, necessity for radiation to bone (for pain or impending fracture), or surgery to bone, because of bone metastases. Sometimes, hypercalcemia of malignancy is also part of the SRE definition.^6^ On the basis of data of a nationwide registry (N = 2052), we have revealed that at diagnosis of metastatic disease, 54% of patients with NSCLC and an *EGFR* mutation (*EGFR+*) have bone metastases, which is the highest incidence compared with 33% in those with *KRAS*+, 31% in those with *ALK* fusion (*ALK*+), and 32% in those with *EGFR/KRAS/ALK* wild type.^7^ Nevertheless, in other mainly small retrospective series (N = 137–209), no differences were observed.^8^^,^^9^

In patients with *EGFR*+ advanced NSCLC, treatment with first- and second-generation EGFR tyrosine kinase inhibitors (TKIs) results in superior progression-free survival (PFS) compared with chemotherapy.^10^ The incidence of SREs in this patient population is high (24%–58%).^1^^,^^11^ In a retrospective series (N = 189), incidence and time to first SRE were similar between patients with *EGFR*+, *KRAS*+, and *EGFR/KRAS* wild-type NSCLC when treated with first-/second-generation EGFR TKI or chemotherapy, respectively.^1^ Nevertheless, patients with *EGFR*+ NSCLC had a significantly longer post-metastatic bone disease survival compared with the other patients (median 15 mo [*EGFR*+], 9.0 mo [*KRAS*+], and 3.2 mo [*EGFR/KRAS* wild type]), (*EGFR+* - *KRAS+*, *p* = 0.049, *EGFR+* - EGFR+*/KRAS+* wildtype, *p* = 0.004).^1^ Consequently, patients with an *EGFR* mutation are longer at risk for new SREs and live longer with SREs, which might affect QoL. Nowadays, osimertinib is the preferred first-line treatment for patients with *EGFR+* NSCLC, with a median PFS of 18.9 months. The prevalence and incidence of SREs during osimertinib treatment are unknown.^12^

Denosumab and bisphosphonates are bone-targeted agents (BTAs) that inhibit normal osteoclast-induced bone resorption. Bisphosphonates are ingested by osteoclasts during bone resorption, which causes cell death of the osteoclast. Denosumab binds to the receptor activator of nuclear factor κB ligand and prevents the interaction with its receptor, receptor activator of nuclear factor κB, with reduction of bone resorption as a result. Both denosumab and bisphosphonates are supposed to have (in)direct antitumor effects, but their precise role has to be elucidated.^13^ BTAs prevent SREs or delay the time to SREs in solid tumors and multiple myeloma.^14^, ^15^, ^16^ Although BTA use in breast cancer is associated with reduction of pain owing to bone metastases, in lung cancer this evidence is less clear, and BTA use is low in patients with lung cancer.^17^, ^18^, ^19^, ^20^, ^21^, ^22^

It could be hypothesized that because of the superior efficacy of osimertinib, less bone metastases and consequently less SREs develop during osimertinib therapy, with as a result less need for the use of BTAs. Reporting of prevalence of bone metastases, SREs and bone-specific outcomes in patients with *EGFR+* NSCLC in clinical trials evaluating EGFR TKIs, including osimertinib, is lacking.^11^ Therefore, we performed this multicenter cohort study to evaluate bone metastases-related outcomes in patients treated with osimertinib.

## Materials and Methods

In this multicenter cohort study, data from patients with *EGFR+* NSCLC in two tertiary referral university hospitals and one teaching hospital in the Netherlands (Maastricht University Center+ [MUMC+], Erasmus Medical Center Cancer Institute [Erasmus MC], and Amphia Hospital) were analyzed.

### Patient Selection and Data Collection

In MUMC+, all patients with metastatic *EGFR+* NSCLC treated with osimertinib as part of regular care between February 2, 2016, and September 22, 2021, were identified using dispensing data from the pharmacy. In Erasmus MC, all patients with metastatic *EGFR+* NSCLC treated with osimertinib between January 18, 2017, and September 22, 2021, were retrieved from a prospective cohort study (START-TKI, NCT05221372). Patients were excluded if no follow-up data were available (at least one follow-up visit after initiation of osimertinib was required).

The inpatient and outpatient medical records of all patients were retrieved. The following data were collected: demographics, date of diagnosis of metastatic NSCLC, smoking status, pathological subtyping of NSCLC, mutational status, presence of bone metastasis at diagnosis of metastatic NSCLC and development of bone metastases during the course of the disease, date of initiation of osimertinib treatment including treatment line, duration of osimertinib treatment and date of progression on osimertinib, presence of SREs in patients with confirmed bone metastases on imaging and if applicable date and type of first SRE, use of bone-targeted agents, and date of death or last follow-up. SREs were defined as either the occurrence of a pathologic fracture, spinal cord compression, necessity for radiation to bone (for pain or impending fracture), surgery to bone because of bone metastases, and hypercalcemia (in patients with bone metastases). SRE at diagnosis of bone metastases was defined as an SRE within 2 months before and 2 months after diagnosis of bone metastases. SRE at initiation of osimertinib was defined as an SRE within 2 months before and 2 months after initiation of osimertinib. Dispensing data from the pharmacy were used to evaluate BTA prescription. Standard radiological evaluation was performed every 2 to 3 months by chest and upper abdomen computer tomography (CT) scans with iodine contrast. The last date of follow-up was October 1, 2021.

Medical ethical committee approval was obtained in accordance with local regulations (METC: 2021-2989 and START-TKI, MEC 2016-643, NCT05221372). The ethics committee waived the need for informed consent for 2021-2989, for the START-TKI study all patients provided informed consent.

### Statistical Analysis

Patient demographics and baseline characteristics are summarized using descriptive statistics. Categorical variables were compared using chi-square tests or Fisher exact probability tests, and continuous variables were compared using the Mann-Whitney *U* test, Kruskal-Wallis test, or analysis of variance. Cox regression analysis was used for univariate and multivariate analyses. The cumulative incidence function, taking the competing risk of mortality into account, was used to calculate the cumulative incidence of bone progression. Survival analysis was performed by Kaplan-Meier analysis. Statistical analyses were performed using SPSS (IBM statistics, version 20).

## Results

### Patient Characteristics

All patients treated with osimertinib (n = 64) in MUMC+ were included. In addition, 186 patients treated with osimertinib from Amphia Hospital and Erasmus MC were enrolled in the START-TKI study. As a result, 250 patients were included in this analysis. Patient characteristics are found in Table 1.

**Table 1:** Patient Characteristics

Characteristics	Total (N = 250)	First-Line Osimertinib (n = 107)^a^	≥Second-Line Osimertinib (n = 143)^a^^,^^b^	*p* Value
Female, n (%)	165 (67)	71 (66)	94 (66)	NS
Never smoker, n (%)	100 (40)	44 (41)	56 (39)	NS
Mean age at diagnosis metastatic NSCLC, y (range)	65.1 (33–87)	67.2 (37–87)	63.6 (33–84)	<0.05
WHO-PS				NS
0–1	180 (72)	80 (75)	100 (70)	
>2	54 (22)	26 (24)	28 (20)	
Unknown	16 (6)	1 (1)	15 (11)	
EGFR mutation				<0.001
Exon 19 deletion	60 (24)	57 (53)	3 (2)	
Exon 21 L858R	28 (11)	25 (23)	3 (2)	
Two mutations simultaneously	8 (3)	7 (7)	1 (1)	
Uncommon	17 (7)	16 (15)	1 (1)	
Original exon 19 deletion or L858R and exon 20 T790M mutation	129 (52)	1 (1)	127 (88)	
Original uncommon and exon 20 T790M mutation	8 (3)	1 (1)	8 (6)	

exon 21 L858R, single-point mutation that substitutes leucine for arginine at position 858 in exon 21; NS, not statistically significant; T790M, point mutation that substitutes methionine for threonine at position 790 in exon 20; TKI, tyrosine kinase inhibitor; WHO-PS, WHO—performance score.

a Percentages were calculated by subgroup.

b All patients received first- or second-generation EGFR TKIs. A total of 123 patients received osimertinib as second-line treatment.

Median follow-up from diagnosis of metastatic NSCLC was 43.0 months (95% confidence interval [CI]: 38.8–47.3 mo). Median follow-up from initiation of osimertinib was 23.4 months (95% CI: 19.9–26.9 mo). In 107 of 250 patients (43%), osimertinib was administered as a first-line treatment.

### Bone Metastases

**Figure 1:**
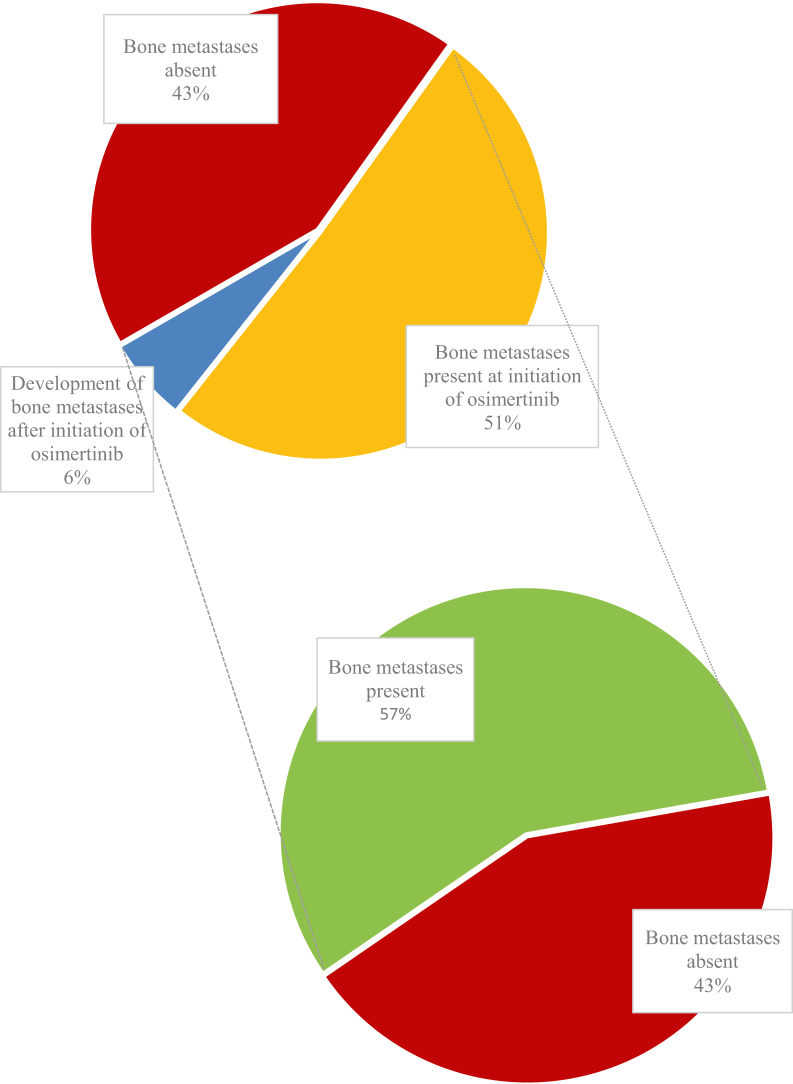
Presence of bone metastases. Time frame of development of bone metastases during NSCLC disease course.

In total, 112 of 250 patients (45%) had synchronous bone metastases at diagnosis of metastatic NSCLC. Of 250 patients, 15 (6%) developed bone metastases before initiation of osimertinib treatment. As a result, 127 of 250 patients (51%) were already diagnosed with having bone metastases at initiation of osimertinib (Fig. 1). Thereafter, 15 of 250 patients (6%) developed bone metastases (14 during and one after osimertinib treatment), resulting in a total of 142 patients (57%) of the whole study population being diagnosed with having bone metastases at the last follow-up.

Of the 250 patients, 25 (10%) developed bone progression or new bone metastases during osimertinib treatment with a median time to event of 6.4 months (95% CI: 2.3–10.6 mo). In three patients, this was the first diagnosis of bone metastases. The cumulative incidence of bone progression at 1 year after diagnosis of stage IV NSCLC was 4.3% (95% CI: 2.2–7.5) and increased to 16.1% (95% CI: 10.9–22.2) at 5 years after diagnosis of stage IV NSCLC. The cumulative incidence of bone progression at 1 year after initiation of osimertinib was 8.8% (95% CI: 5.5–12.9) and increased to 14.2% (95 CI: 9.4–19.9) at 5 years after initiation of osimertinib.

### Skeletal-Related Events

**Figure 2:**
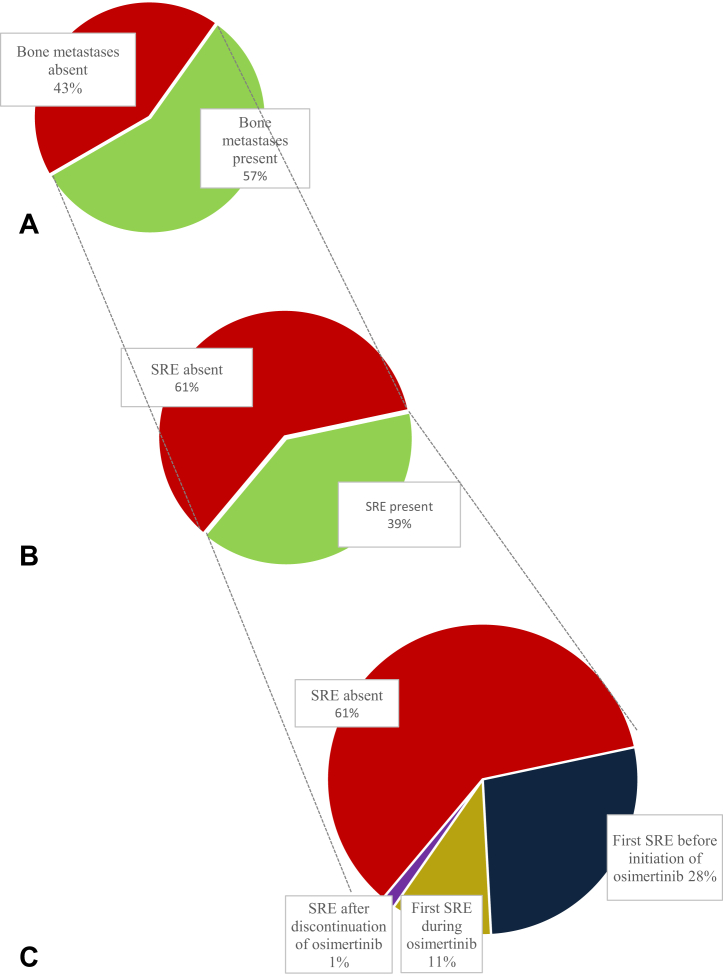
Presence of skeletal-related events. (*A*) Bone metastases during NSCLC disease course. (*B*) Presence of SRE in patients with bone metastases. (*C*) Time frame of SRE development in patients with bone metastases during NSCLC disease course. SREs are presented as percentage of the study population with bone metastases, for example, 39 patients have an SRE before initiation of osimertinib. SRE, skeletal-related event.

Of the 142 patients with bone metastases, 21 (15%) presented with a first SRE at diagnosis of metastatic NSCLC, and in total, 56 patients (40%) developed one or more SREs during the course of their disease. Furthermore, 28% of the patients developed their first SRE before, 11% during osimertinib treatment, and 1% after discontinuation of treatment (Table 2 and Fig. 2). The median time to first SRE for patients who did not have an SRE at metastatic NSCLC diagnosis was 10.1 months (95% CI: 6.9–13.3 mo). In the group of patients with the first SRE during osimertinib treatment (15 of 56 patients), the median time to SRE was 4.8 months (95% CI: 2.1–7.6 mo).

### Survival Outcomes

The median PFS from initiation of osimertinib was 16.5 months (95% CI: 14.2–18.9 mo) for the total study population. Although numerically higher, there was no significant difference in median PFS between patients treated with osimertinib in first line or in second line and beyond (median PFS of 18.9 mo [95% CI: 14.3–23.5 mo] versus 16.3 mo [95% CI: 14.5–18.1 mo]; *p* = 0.575, respectively).

At data cutoff, 106 of 250 patients (42%) had deceased. The median OS from diagnosis of metastatic NSCLC was 48.5 months (95% CI: 39.8–57.2) and was significantly shorter for patients with bone metastases during the course of their disease than for those without: 37.2 months (95% CI: 33.3–41.1) versus 66.6 months (95% CI: 55.9–77.2) (*p* < 0.0001, hazard ratio [HR] = 2.4 [95% CI: 1.6–3.6]). The median OS for patients with bone metastases and a minimum of one SRE was not significantly different compared with those without SREs: 41.1 months (95% CI: 27.3–54.9) versus 36.5 months (95% CI: 29.4–43.5) (*p* = 0.585, HR = 1.1 [95% CI: 0.7–1.8]). Multivariate analysis revealed uncommon mutations, presence of bone metastases and bone progression, or development of new bone metastases during osimertinib treatment as independent negative prognostic factors for OS (*p* = 0.009, *p* = 0.001, and *p =* 0.02, respectively) (Table 3).

**Table 2:** Bone Metastases- and Bone-Related Outcomes

Characteristics	Total (N = 250)	First-Line Osimertinib (n = 107)^a^	≥Second-Line Osimertinib (n = 143)^a^^,^^b^	*p* Value
Bone metastases at diagnosis stage IV, n (%)	112 (45)	55 (51)	57 (40)	NS
Bone metastases at initiation of osimertinib, n (%)	127 (51)	56 (52)	71 (50)	NS
New bone metastases or bone progression during osimertinib, n (%)	25 (10)	10 (10)	15 (11)	NS
Presence of minimum one SRE in patients with NSCLC with bone metastases, n (%)	56 (40)	22 (36)	34 (42)	<0.05
First SRE at diagnosis of NSCLC in patients with NSCLC with bone metastases^c^	19 (13)	11 (8)	8 (6)	<0.05
First SRE before initiation of osimertinib in patients with NSCLC with bone metastases^c^^,^^d^	20 (28)	1 (9)	19 (19)	NS
First SRE during osimertinib in patients with NSCLC with bone metastases^c^	15 (11)	8 (6)	7 (5)	NS
Type of first SRE, n (%)^a^				<0.05
Radiotherapy	45 (80)	17 (30)	28 (50)	
Pathologic fracture due to bone metastasis	4 (7)	2 (4)	2 (4)	
Surgery	6 (11)	3 (5)	3 (5)	
Spinal cord compression	1 (2)	0 (0)	1 (2)	
BTA use in patients with bone metastases*,* n (%)^c^	23 (16)	5 (4)	18 (13)	NS

BTA, bone-targeted agent; NS, not statistically significant; SRE, skeletal-related event; TKI, tyrosine kinase inhibitor.

a Percentages were calculated by subgroup.

b All patients received first- or second-generation EGFR TKIs. A total of 123 patients received osimertinib as second-line treatment.

c Percentages were calculated by all patients with bone metastases (n = 142).

d Numbers were calculated minus patients with SRE at diagnosis of advanced NSCLC. For example, one patient developed an SRE between diagnosis of advanced NSCLC and initiation of osimertinib.

**Table 3:** Univariate and Multivariate Analyses for Overall Survival From Diagnosis of Stage IV NSCLC

Variables	Univariate Analysis		Multivariate Analysis	
	HR (95% CI)	*p* Value	HR (95% CI)	*p* Value
Sex			-	-
Female	1 (reference)			
Male	1.33 (0.89–1.98)	0.160		
Smoke			-	-
Never	1 (reference)			
Current	1.24 (0.44–3.51)	0.681		
Former	1.67 (1.11–2.53)	0.015^a^		
EGFR mutation				
Exon 19 deletion and exon 20 T790M mutation	1 (reference)		1 (reference)	
Exon 21 L858R and exon 20 T790M mutation	1.62 (1.05–2.48)	0.028^a^	1.54 (0.99–2.40)	0.057
Two mutations simultaneously	2.39 (0.74–7.75)	0.146	1.59 (0.46–5.51)	0.462
Uncommon and exon 20 T790M mutation	2.57 (1.45–4.55)	0.001^a^	2.26 (1.23–4.17)	0.009^a^
TKI line				
First line	1 (reference)		1 (reference)	
Second line	0.63 (0.40–1.00)	0.048^a^	0.71 (0.43–1.19)	0.196
Mean age at diagnosis metastatic NSCLC	1.00 (0.98–1.02)	0.825	-	
Bone metastasis				
Absent	1 (reference)		1 (reference)	
Present	2.39 (1.57–3.65)	<0.001^a^	2.32 (1.43–3.74)	0.001^a^
Bone progression or new bone metastases during osimertinib				
Absent	1 (reference)		1 (reference)	
Present	2.42 (1.48–4.0)	<0.001^a^	1.93 (1.11–3.35)	0.020^a^
Skeletal-related event				
Absent	1 (reference)			
Present	1.42 (0.93–2.16)	0.103	0.69 (0.42–1.15)	0.155
Bone-targeted agent use in patients with bone metastases			-	-
Absent	1 (reference)			
Present	1.22 (0.70–2.12)	0.485		

HR, hazard ratio; CI, confidence interval; exon 21 L858R, single-point mutation that substitutes leucine for arginine at position 858 in exon 21; T790M, point mutation that substitutes methionine for threonine at position 790 in exon 20; TKI, tyrosine kinase inhibitor.

a Data indicate a *p* < 0.05.

**Figure 3:**
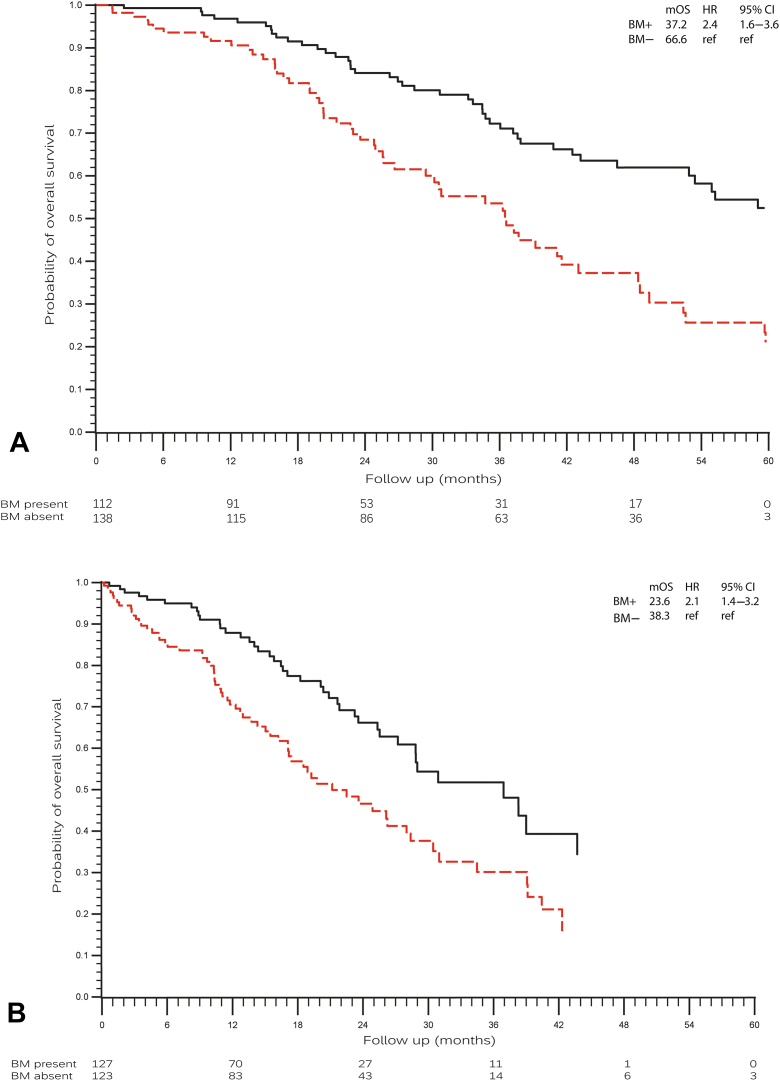
(*A*) Overall survival from diagnosis of metastatic NSCLC. Black line: patients with metastatic NSCLC without bone metastases; red dashed line: patients with metastatic NSCLC with bone metastases. (*B*) Overall survival from initiation of osimertinib. Black line: patients with metastatic NSCLC without bone metastases; red dashed line: patients with metastatic NSCLC with bone metastases. BM−, bone metastases absent; BM+, bone metastases present; CI, confidence interval; HR, hazard ratio; mOS, median overall survival; ref, reference.

The median OS from initiation of osimertinib treatment was 28.0 months (95% CI: 23.8–32.2) and was significantly shorter for patients with bone metastases than for patients without bone metastases during the course of their disease: 23.6 months (95% CI: 17.1–30.0) versus 38.3 months (95% CI: 23.9–52.7) for patients without bone metastases (*p* < 0.0001, HR 2.1 [95% CI:1.4–3.2]). The median OS for patients with bone metastases and a minimum of one SRE was not significantly different compared with those without SREs: 26.1 months (95% CI: 18.2–34.1) versus 22.5 months (95% CI: 14.7–30.3) (*p* = 0.939, HR 1.0 [95% CI: 0.6–1.6]). In Figure 3*A* and *B*, the median OS for the study population with/without bone metastases, subdivided by the different treatment lines, is illustrated. The median OS after development of bone metastasis was 30.8 months (95% CI: 21.9–39.7). The median OS after development of the first SRE was 31.1 months (95% CI: 15.8–46.5).

## Discussion

Baseline and cumulative incidence of bone metastases and SREs is high in patients with *EGFR+* metastatic NSCLC treated with first- and second-generation EGFR TKIs, and therefore, better treatment options are necessary.^11^ We found that most patients (45%) already had bone metastases at first diagnosis of metastatic NSCLC, and this percentage increased to 51% at initiation of osimertinib if patients were treated with osimertinib in second line and beyond. At diagnosis of metastatic *EGFR+* NSCLC, 15% of patients with bone metastases were diagnosed with having an SRE, and the cumulative incidence increased to 39%. Consequently, both prevention of progression of existing bone metastases and SREs and prevention of new events are important. We found that during osimertinib treatment, 10% of the patients developed new bone metastases or progression of existing bone metastases. In other series (including a systematic review) evaluating EGFR TKI trials (N = 1196) and several retrospective series evaluating patients (N = 126–1081) treated with EGFR TKI, the percentage of patients with bone metastases at diagnosis of metastatic NSCLC was similar to our study (Supplementary Table 1).^11^^,^^23^ Nevertheless, data about bone progression and development of SREs during EGFR TKI treatment are scarce.^11^ The percentage of patients who develop bone progression during osimertinib in our series is comparable with that of a smaller series (N = 126) evaluating outcomes on first-line osimertinib (10% versus 12%) and with trials evaluating first- and second-generation EGFR TKIs (11% versus 3%–26%) (Supplementary Table 1).^23^, ^24^, ^25^, ^26^, ^27^, ^28^, ^29^, ^30^, ^31^, ^32^, ^33^ The highest percentages of bone progression were found in two studies (N = 38–53) in which regularly a 2-deoxy-2-[fluorine-18] fluoro-D-glucose positron emission tomography-computed tomography scan (FDG-PET-CT scan) was made during follow-up. This is not surprising as FDG-PET has a high sensitivity to detect bone metastases.^24^^,^^26^^,^^34^ Another small series (N = 101) in patients treated with osimertinib in second line (78% of patients) and beyond also reported a 22% bone progression rate. Radiological tumor assessment during follow-up was comparable with that of our series.^35^

We are the first to report the incidence of SREs during osimertinib treatment (11% of the patients with bone metastases developed their first SRE during osimertinib treatment), which is more than half compared with the 25.9% to 28% observed in series (N = 274–552) evaluating first- or second-generation EGFR TKI.^4^^,^^36^

In our series, we report a relatively long median OS of 48.5 months, and although shorter, most patients with bone metastases survived more than 3 years (median OS = 37.2 mo). Development of SREs did not significantly impair OS (median OS after first SRE was 41.1 mo vs 36.5 mo in patients without SREs, *p* = 0.585). As our population consists of a mixture of treatment-naive patients and pretreated patients, other patient and/or tumor characteristics (e.g., more resistant tumor cells, older age, increased WHO performance score) could also influence OS. Most SREs occurred already at diagnosis or developed during the first year after a diagnosis of bone metastases. Previous studies revealed that SREs have an impact on patient-reported outcomes with a decline in patients’ physical and emotional well-being, ability to perform basic functions of daily living, and QoL.^37^^,^^38^ Furthermore, we know that previous SREs are a risk factor for development of new SREs and patients with *EGFR*-mutated NSCLC have a long post-metastatic bone disease survival.^1^^,^^39^ That is why any reduction in SREs, even if it does not lead to improvement in OS, is important too. BTAs are not specifically recommended in Dutch NSCLC or bone metastases guidelines.^40^^,^^41^ In clinical practice, BTAs are not frequently used in the treatment strategy of NSCLC, as is also reflected in the low percentage of use (only 16% in patients with bone metastases) in our series. Data are also lacking on BTA use in other series evaluating *EGFR*+ NSCLC. In series (N = 114–10,982) evaluating patients with NSCLC unselected for oncogenic drivers, uptake of BTA use was also limited (15%–38%).^19^, ^20^, ^21^ This low BTA use is in contrast with the European Society for Medical Oncology clinical practice guideline on bone health in which it is recommended to start a BTA in most patients as soon as bone metastases are diagnosed, whether they are symptomatic or not.^13^ In metastatic breast and prostate cancers, two solid malignancies with a similar favorable prognosis as *EGFR*+ NSCLC, most patients with bone metastases received a BTA, which translated into a significant SRE reduction by bisphosphonates in patients with breast cancer and bone metastases (relative risk = 0.86, 95% CI: 0.78–0.95, *p* = 0.003).^14^^,^^21^

On the basis of our data and the international guideline recommendations, we strongly recommend to prospectively evaluate and consider the use of BTA (as in daily practice they are barely used) in this specific oncogenic-driven subgroup with a favorable survival, also post-bone metastases diagnosis, to reduce the burden of SREs.^42^^,^^43^ Other arguments for the use of BTA are small, hypothesis generating, in vivo (N = 62–129) and in vitro series that reveal synergy between bisphosphonates and EGFR TKIs with effects on tumor suppression, PFS, and OS post-bone metastases.^44^, ^45^, ^46^ This synergistic effect should be evaluated prospectively. Currently, one trial (NCT03958565) is enrolling patients with bone metastasized NSCLC to evaluate the percentage reduction of bone markers in urine or serum while treated with zoledronic acid or denosumab. This study population is subdivided in patients with any oncogenic driver treated with a TKI and in patients without actionable mutations treated with chemotherapy and/or immunotherapy. The incidence of SREs in both groups is a secondary outcome measurement.

This study has its limitations. First, part of the data was retrospectively collected. Nevertheless, bone metastases and SREs are relevant clinical events that are captured in the medical records. Second, not all patients underwent a FDG-PET-CT scan or bone scintigraphy to detect asymptomatic bone metastases, and we did not have detailed information on location and burden of bone metastases. Nevertheless, there was no underreporting of SREs as these per definition cause complaints. Third, we included all lines of osimertinib treatment as although osimertinib is the preferred first-line treatment, not all patients worldwide have access to first-line osimertinib, and data on osimertinib in second line and beyond remain therefore important.^47^^,^^48^ Finally, as it was a retrospective study, we could not evaluate the impact of SREs on patient-reported outcomes. Nevertheless, other studies already revealed the impact of SREs on patients’ QoL.^37^^,^^38^

To conclude, bone metastases and SREs are frequent events both before and during treatment with osimertinib in patients with *EGFR*+ NSCLC. These findings together with the long OS after the occurrence of bone metastases and SREs advocate the prescription of BTAs in *EGFR*+ NSCLC with bone metastases and the use of bone-specific end points in clinical trials.

## CRediT Authorship Contribution Statement

**Anita J. W. M. Brouns:** Conceptualization, Methodology, Validation, Formal analysis, Investigation, Writing—original draft, Visualization.

**Ard van Veelen:** Validation, Writing—review and editing, Visualization.

**Marijn Veerman:** Validation, Resources, Writing—review and editing,

**Christi Steendam:** Resources, Writing—review and editing.

**Safiye Dursun:** Resources, Writing—review and editing.

**Cor van der Leest:** Resources, Writing—review and editing.

**Sander Croes:** Writing—review and editing, Supervision.

**Anne-Marie C. Dingemans:** Conceptualization, Methodology, Resources, Writing—review and editing, Supervision.

**Lizza E. L. Hendriks:** Conceptualization, Resources, Writing—review and editing, Supervision.
