# Supplementary material for: Incidence of Bone Metastases and Skeletal-Related Events in Patients With EGFR-Mutated NSCLC Treated With Osimertinib
Source: JTO Clin Res Rep. 2023 Apr 3;4(5):100513. doi: 10.1016/j.jtocrr.2023.100513 (PMC10165134; doi:10.1016/j.jtocrr.2023.100513)
Supplement: Supplementary Table [file mmc1.docx]

**Supplementary material**

**Table S1 – Summary of reported bone metastases and SREs of EGFR-TKI studies.**

| **Study (y)** | **Trial type** | **Total pts/*EGFR+* pts** | **Treatment arm dose (% of treatment arm)** | **Comparator arm dose  (% of treatment arm)** | **Median follow-up (months)** | **BM at baseline n (%)** | **Number of pts with BM progression of total pts with PD n (%)** | **Number of pts with BM progression of total study population n (%)** | **SRE at baseline in pts with BM  n (%)** | **SRE during treatment**  **in pts with BM n (%)** |
| --- | --- | --- | --- | --- | --- | --- | --- | --- | --- | --- |
| Sunaga (2007)^1^ | Phase II, single-arm, multicenter study | 21/21 | Gefitinib 250 mg q.d. (100) | - | 12.6 | 5/21 (24) | NR | NR | NR | NR |
| Inoue (2009)^2^ | Phase II, single-arm study | 29/29 | Gefitinib 250 mg q.d. (100) | - | 17.8 | 12/29 (41) | NR | NR | NR | NR |
| Rosell (2012)  [Eurtac]^3^ | Phase III, open-label, multicenter RCT | 173/173 | Erlotinib 150 mg q.d. (50) | 3-week cycles of chemotherapy^1^ (50) | Erlotinib arm: 18.9 Chemotherapy arm: 14.4 | Erlotinib arm: 28/86 (33)  Chemotherapy arm: 29/87 (33) | NR | NR | NR | NR |
| Yoshimura (2013)^4^ | Phase II, single-arm, study | 27/27 | 3-weekly cycles of pemetrexed d1 500mg/m^2^ and erlotinib/gefitinib d2-16 dose NR (100) | - | 11.4 | 16/27 (59) | NR | NR | NR | NR |
| Reguart (2014)^5^ | Phase I-II, single-arm, multicenter study | 25/25 | Erlotinib 150mg q.d. + vorinostat 400mg q.d. (100) | - | NR | 10/25 (40) | NR | NR | NR | NR |
| Zwitter (2014)^6^ | Phase II, single-arm, study | 53/38 | 3-weekly cycles of gemcitabin 120mg/m^2^ d1, cisplatin 75mg/m^2^ d2, gemcitabin 1250mg/m^2^ d4, erlotinib 150mg q.d. d5-15 (100) | - | NR | 24/38 (63) | *EGFR+* group: “bone (10) most frequent site of PD.” Number of pts with PD NR | *EGFR+* group: 10/38 (26) | NR | NR |
| Yoshimura (2015)^7^ | Phase II, open-label, single-arm study | 26/26 | 3-weekly cycles of pemetrexed d1 500mg/m^2^ and gefitinib 250mg q.d. d2-16 (100) | - | 19.7 | 8/26 (31) | NR | NR | NR | NR |
| Park (2016a)  [Aspiration study]^8^ | Phase II, single-arm, multicenter  study | 207/207 | Erlotinib 150mg q.d. (100) | - | 11.3 | NR | 14/171 (8) | 14/207 (21) | NR | NR |
| Park (2016b)  [Lux-lung 7]^9^ | Phase IIB, open-label, multicenter RCT | 319/319 | Afatinib 40mg q.d.; dose escalation to 50mg q.d. allowed after 4 weeks without AE (50) | Gefitinib 250mg q.d. (50) | 27.3 | Afatinib arm: 80/160 (50)  Gefitinib arm: 73/159 (46) | NR | NR | NR | NR |
| Zwitter (2016)^10^ | Phase II, open-label, single-arm, study | 38/38 | 3-weekly cycles of gemcitabin 1250mg/m^2^ d1+4, cisplatin 75mg/m2 d2, erlotinib 150mg q.d. d 5-15 (100) | - | 35 | 24/38 (63) | “Bone (10) most frequent site of PD.” Number of pts with PD NR. | 10/38 (26) | NR | NR |
| Atagi (2016)^11^ | Combined results of 2 phase II studies: JO22903 (single arm) and JO25567 study (randomized) | 177/177 | JO22903: erlotinib 150mg q.d. (56)  JO25567: erlotinib 150mg q.d. (22) | JO22903: -  JO25567: bevacizumab 15mg/kg 3-weekly  cycles + erlotinib 150mg q.d. (22) | JO22903: 20.4  JO25567: at  minimum 20 | NR | 20/125 (16) | 20/177 (11) | NR | NR |
| Hirano (2016)^12^ | Phase II, single-arm, multicenter study | 11/11 | Erlotinib 25mg q.d.; dose escalation to 150mg q.d. in case of PD (100) | - | NR | NR | 1/8 (13) | 1/11 (9) | NR | NR |
| Goss (2016)  [Aura 2]^13^ | Phase II, open-label, multicenter single-arm study | 199/199 | Osimertinib 80 mg q.d. (100) | - | 13.0 | NR | 9/65 (14) | 9/199 (5) | NR | NR |
| Mok (2017)  [Aura 3]^14^ | Phase III, open-label, multicenter RCT | 419/419 | Osimertinib 80mg q.d. (67) | 3-weekly cycles of pemetrexed 500mg/m^2^ + carboplatin AUC 5 or cisplatin 75mg/m^2^ (33) | 8.3 | NR | Osimertinib arm: 9/97 (9)  Platinum/ pemetrexed arm: 6/101 (6) | Osimertinib arm: 9/277 (3) Platinum/ pemetrexed arm: 6/149 (4) | NR | NR |
| Soria (2018)  [Flaura]^15^ | Phase III, multicenter, double-blind, RCT | 556/556 | Osimertinib 80mg q.d. (50) | Erlotinib 150mg q.d. or Gefitinib 250mg q.d. (50) | 15 | NR | Osimertinib arm: 11/NR  Gefitinib or erlotinib arm: 11/NR | Osimertinib arm: 11/278 (4)  Gefitinib or erlotinib arm: 11/278 (4) | NR | NR |
| Lim (2018)^16^ | Phase II, single-arm, study | 49/49 | Gefitinib 250mg q.d. (100) | - | At minimum 6 | 9/49 (18) | NR | NR | NR | NR |
| Ahn (2019)^17^ | Combined results of 2 phase II studies (AURA extension and AURA 2 trial), both single arm | 411/411 | Osimertinib 80mg q.d. (100) | - | NR | NR | 28/NR | 28/411 (7) | NR | NR |
| Zheng (2019)^18^ | Phase II, single-arm study | 10/10 | Erlotinib 150mg q.d. or Gefitinib 250mg q.d.  plus thoracic radiotherapy^4^ (100) | - | 12 | 9/10 (90) | 2/7 (29) | 2/10 (20) | NR | NR |
| Cho (2019)  [KCSG-Lu15-09]^19^ | Phase II, open-label, single arm, study | 36/36 | Osimertinib 80mg q.d. (100) | - | 20.6 | 10/36 (28) | NR | NR | NR | NR |
| Noronha (2020)^20^ | Phase III, open-label, study | 350/350 | 3-weekly cycles of Gefitinib 250mg q.d. and pemetrexed 500mg/m^2^ + carboplatin AUC 5 on d1, (up to four  cycles), followed by  3-weekly cycles  maintenance pemetrexed (50) | Gefitinib 250mg q.d. (50) | 17 | Gefitinib+ chemo arm: 24/174 (14)  Gefitinib arm: 25/176 (14) | Gefitinib + chemo arm: 3/97 (3)  Gefitinib arm: 7/136 (5) | Gefitinib + chemo arm: 3/175 (2)  Gefitinib arm: 7/175 (4) | NR | NR |
| Wu (2020)  [Insight study]^21^ | Phase Ib/II, open-label, study | 55/55 | Teponitinib 500mg q.d. + gefitinib 250mg q.d. (66) | Pemetrexed 500mg/m^2^+ cisplatin 75mg/m^2^ or carboplatin AUC 5-6 on d1 ≤6 cycles or 4  cycles + pemetrexed maintenance (34) | 21.8 | Teponitinib plus gefitinib arm: 15/49 (23)  Chemotherapy arm: 9/24 (38) | NR | NR | NR | NR |
| Lagana (2020)^22^ | Retrospective multicenter study | 274/274 | First-line gefitinib 250mg q.d. (67), erlotinib 150mg q.d. (16), afatinib 40mg q.d. (17), osimertinib 80mg q.d. (0.4) | - | 23 | 274/274 (100) | NR | NR | 77/274 (28) | NR |
| Luo (2021)^23^ | Prospectively observed cohort study | 417/417^3^ | ≥2nd line osimertinib 80mg q.d. (100) | - | 49.2 | 76/154 (49)^5^ | NR | NR | NR | NR |
| Dal Maso (2021)^24^ | Retrospective multicenter study | 139/139 | ≥2nd line osimertinib 80mg q.d. (73), any systemic treatment (27) | - | 14.1 | Osimertinib arm: 46/101 (46) Any systemic treatment arm: 8/38 (21) | Osimertinib arm: 22/71 (31) Any systemic treatment arm: 6/33 (18) | Osimertinib arm: 22/101 (22) Any systemic treatment arm: 6/21 (29) | NR | NR |
| Lorenzi (2021)^25^ | Real-world. Prospective study | 126/126 | First-line osimertinib 80mg q.d. (100) | - | 12.3 | 59/126 (47) | 15/44 (34) | 15/126 (12) | NR | NR |
| Gen (2022)^26^ | Retrospective cohort study | 388/388 | First-line gefitinib 250mg q.d./erlotinib 150mg q.d. (47), afatinib 40mg q.d. (14), osimertinib 80mg q.d. (39) | - | NR | 160/388 (41) | NR | NR | NR | NR |
| Zeng (2022)^27^ | Retrospective cohort study | 1081/1081 | First-line gefitinib 250mg q.d./erlotinib 150mg q.d. (86), afatinib 40mg q.d. (5), osimertinib 80mg q.d. (9) | - | 35 | 485/1081 (45) | 58/619 (9) | 58/1081 (5) | NR | NR |

Abbreviations: SREs; skeletal related events, EGFR-TKI; epidermal growth factor receptor tyrosine kinase inhibitors, y; year, pts; patients, EGFR+; activating mutation in the epidermal growth factor receptor (EGFR), BM; bone metastasis, q.d.; once a day, NR; not reported, RCT; randomized controlled trial, AE; adverse events, AUC; area under the curve.

^1^ Cisplatin 75 mg/m² on day 1 plus docetaxel (75 mg/m² on day 1) or gemcitabin (1250 mg/m² on days 1 and 8). In patients with contra-indications for cisplatin, carboplatin (AUC 6 with docetaxel 75 mg/m² or AUC 5 with gemcitabin 1000 mg/m²) was allowed.
^3^ Only 154 out of 417 patients received 1^st^/2^nd^ generation TKI with subsequent osimertinib, the other 263 patients received 1^st^/2^nd^ generation TKI without subsequent osimertinib (n=203) or no EGFR-TKI treatment (n=60)
^4^ 54-60 Gray / 27-30 fractions / 5.5-6 weeks.
^5^ Percentage bone metastases at initiation of osimertinib, percentage at baseline is not reported.

**References**

1. Sunaga N, Tomizawa Y, Yanagitani N, et al. Phase II prospective study of the efficacy of gefitinib for the treatment of stage III/IV non-small cell lung cancer with EGFR mutations, irrespective of previous chemotherapy. *Lung Cancer* 2007;56:383-389.

2. Inoue A, Kobayashi K, Usui K, et al. First-line gefitinib for patients with advanced non-small-cell lung cancer harboring epidermal growth factor receptor mutations without indication for chemotherapy. *Journal of Clinical Oncology* 2009;27:1394-1400.

3. Rosell R, Carcereny E, Gervais R, et al. Erlotinib versus standard chemotherapy as first-line treatment for European patients with advanced EGFR mutation-positive non-small-cell lung cancer (EURTAC): A multicentre, open-label, randomised phase 3 trial. *The Lancet Oncology* 2012;13:239-246.

4. Yoshimura N, Okishio K, Mitsuoka S, et al. Prospective assessment of continuation of erlotinib or gefitinib in patients with acquired resistance to erlotinib or gefitinib followed by the addition of pemetrexed. *Journal of Thoracic Oncology* 2013;8:96-101.

5. Reguart N, Rosell R, Cardenal F, et al. Phase I/II trial of vorinostat (SAHA) and erlotinib for non-small cell lung cancer (NSCLC) patients with epidermal growth factor receptor (EGFR) mutations after erlotinib progression. *Lung Cancer* 2014;84:161-167.

6. Zwitter M, Stanic K, Rajer M, et al. Intercalated chemotherapy and erlotinib for advanced NSCLC: High proportion of complete remissions and prolonged progression-free survival among patients with EGFR activating mutations. *Radiology and Oncology* 2014;48:361-368.

7. Yoshimura N, Kudoh S, Mitsuoka S, et al. Phase II study of a combination regimen of gefitinib and pemetrexed as first-line treatment in patients with advanced non-small cell lung cancer harboring a sensitive EGFR mutation. *Lung Cancer* 2015;90:65-70.

8. Park K, Yu CJ, Kim SW, et al. First-line erlotinib therapy until and beyond response evaluation criteria in solid tumors progression in Asian patients with epidermal growth factor receptor mutation-positive non-small-cell lung cancer the ASPIRATION study. *JAMA Oncology* 2016;2:305-312.

9. Park K, Tan EH, O'Byrne K, et al. Afatinib versus gefitinib as first-line treatment of patients with EGFR mutation-positive non-small-cell lung cancer (LUX-Lung 7): A phase 2B, open-label, randomised controlled trial. *The Lancet Oncology* 2016;17:577-589.

10. Zwitter M, Rajer M, Stanic K, et al. Intercalated chemotherapy and erlotinib for non-small cell lung cancer (NSCLC) with activating epidermal growth factor receptor (EGFR) mutations. *Cancer Biology and Therapy* 2016;17:833-839.

11. Atagi S, Goto K, Seto T, et al. Erlotinib for Japanese patients with activating EGFR mutation-positive non-small-cell lung cancer: Combined analyses from two Phase II studies. *Future Oncology* 2016;12:2117-2126.

12. Hosomi Y, Morita S, Sugawara S, et al. Gefitinib alone versus gefitinib plus chemotherapy for non–small-cell lung cancer with mutated epidermal growth factor receptor: NEJ009 study. *Journal of Clinical Oncology* 2020;38:115-123.

13. Goss G, Tsai CM, Shepherd FA, et al. Osimertinib for pretreated EGFR Thr790Met-positive advanced non-small-cell lung cancer (AURA2): a multicentre, open-label, single-arm, phase 2 study. *The Lancet Oncology* 2016;17:1643-1652.

14. Mok TS, Wu Y-L, Ahn M-J, et al. Osimertinib or Platinum–Pemetrexed in EGFR T790M–Positive Lung Cancer. *New England Journal of Medicine* 2017;376:629-640.

15. Soria J-C, Ohe Y, Vansteenkiste J, et al. Osimertinib in Untreated EGFR-Mutated Advanced Non–Small-Cell Lung Cancer. *New England Journal of Medicine* 2018;378:113-125.

16. Lim SW, Park S, Kim Y, et al. Continuation of gefitinib beyond progression in patients with EGFR mutation-positive non-small-cell lung cancer: A phase II single-arm trial. *Lung Cancer* 2018;124:293-297.

17. Ahn MJ, Tsai CM, Shepherd FA, et al. Osimertinib in patients with T790M mutation-positive, advanced non–small cell lung cancer: Long-term follow-up from a pooled analysis of 2 phase 2 studies. *Cancer* 2019;125:892-901.

18. Zheng L, Wang Y, Xu Z, et al. Concurrent EGFR‐TKI and Thoracic Radiotherapy as First‐Line Treatment for Stage IV Non‐Small Cell Lung Cancer Harboring EGFR Active Mutations. *The Oncologist* 2019;24:1031-1031.

19. Cho JH, Lim SH, An HJ, et al. Osimertinib for patients with non-small-cell lung cancer harboring uncommon EGFR mutations: A multicenter, open-label, phase II trial (KCSG-Lu15-09). 2020;38:488-495.

20. Noronha V, Patil VM, Joshi A, et al. Gefitinib versus gefitinib plus pemetrexed and carboplatin chemotherapy in EGFR-mutated lung cancer. American Society of Clinical Oncology;38:124-136 Available at <https://pubmed.ncbi.nlm.nih.gov/31411950/>.

21. Wu YL, Cheng Y, Zhou J, et al. Tepotinib plus gefitinib in patients with EGFR-mutant non-small-cell lung cancer with MET overexpression or MET amplification and acquired resistance to previous EGFR inhibitor (INSIGHT study): an open-label, phase 1b/2, multicentre, randomised trial. *The Lancet Respiratory Medicine* 2020;8:1132-1143.

22. Lagana M, Gurizzan C, Roca E, et al. High Prevalence and Early Occurrence of Skeletal Complications in EGFR Mutated NSCLC Patients With Bone Metastases. *Front Oncol* 2020;10:588862.

23. Luo YH, Liu H, Wampfler JA, et al. Real-world efficacy of osimertinib in previously EGFR-TKI treated NSCLC patients without identification of T790M mutation. *J Cancer Res Clin Oncol* 2022;148:2099-2114.

24. Dal Maso A, Lorenzi M, Ferro A, et al. Real-world data on treatment outcomes in EGFR-mutant non-small-cell lung cancer patients receiving osimertinib in second or further lines. *Future Oncol* 2021;17:2513-2527.

25. Lorenzi M, Ferro A, Cecere F, et al. First-Line Osimertinib in Patients with EGFR-Mutant Advanced Non-Small Cell Lung Cancer: Outcome and Safety in the Real World: FLOWER Study. *Oncologist* 2021.

26. Gen S, Tanaka I, Morise M, et al. Clinical efficacy of osimertinib in EGFR-mutant non-small cell lung cancer with distant metastasis. *BMC Cancer* 2022;22:654.

27. Zeng Y, Guo T, Zhou Y, et al. Clinical outcomes of advanced non-small cell lung cancer patients harboring distinct subtypes of EGFR mutations and receiving first-line tyrosine kinase inhibitors: brain metastasis and de novo T790M matters. *BMC Cancer* 2022;22:198.
